# Therapeutical Effects of Turpentine

**Published:** 1850-08

**Authors:** Thomas Smith


*Therapeutical Effects of Turpentine.* By Thomas Smith, M. D.—The diseases for which turpentine has been prescribed, and which have been materially relieved by it, are extremely numerous; there is scarcely one, whether acute or chronic, sthenic or asthenic, which has not been successfully treated, if the testimony of some of the first practitioners of the age is to be credited, by the medicine under consideration. It would be a useless task to cite all the cases and all the maladies in which the admirers of this drug have found it advantageous. Suffice it to say, that in every instance where prejudice has not interfered, and where ignorance has not prescribed, this drug has obtained favor and proved itself a faithful friend.

In passing in review the numerous disorders for which it has been ordered, as I wish this paper to have a practical bearing, I shall dwell as briefly as posible upon all those which have not come under my own immediate observation. Those who desire a more extensive acquaintance with the nature, properties, and uses of this drug than is to be met with in these sketches, will do well to consult the pages of our monthly and weekly periodicals, which, for the last thirty years, have occupied a prominent place in the medical literature of Europe and America. The writings of Dr. Copland, Paris, Pereira, Eberle, Thompson, Brande, etc., the Dictionnaire de la Matière Medicale, and the records of ancient medicine, contain an amount of valuable information regarding the properties of turpentine. In common with other medicines, its therapeutic effects are liable to be modified by numerous circumstances, viz.: the seasons of the year, the idiosyncrasies, age, or sex, of the individual, the special or general cause of the malady, or its occurrence before, or subsequent to, any general or universal epidemic.* From a neglect of these precautions, many really valuable remedies have, though somewhat undeservedly, fallen into disrepute.

As a rapid and safe *counter-irritant*, there is no drug more efficacious than warm oil of turpentine or camphine. I have never known an instance of its acting injuriously when thus applied; it never produces stranguary or any uneasiness of the urinary organs, like preparations of cantharides; and here I fully coincide with the opinion expressed by the late Dr. Ryan, that when counter irritation is deemed imperatively necessary in severe acute diseases,

---

*It is a remarkable fact that after any severe visitation, such as epidemic cholera, the human frame undergoes an extraordinary change. Many will, I have no doubt, recollect how general was the custom to abstract large quantities of blood in fevers and inflammatory disorders previous to 1831. Venesection was the practice of the day. On the advent of the epidemic influenza of 1833, general bleeding, even in maladies of a high phlogistic character, could not be adopted with safety; numerous lives were doubtless sacrificed, ere this change in the human constitution—its inaptitude to bear excessive depletory measures, was fully appreciated and understood. We are now approaching an epoch (if we have not already entered it,) in which the vital phenomena of the animal organism will manifest themselves differently under the influence of remedial agents. If my observation does not deceive me, I am inclined to believe that this great climacteric change, on the completion of the cycle of the late formidable and universal epidemic, will mainly develop itself, by inducing a lax condition of the intestinal tube. I have noticed, that patients who have been accustomed to take large quantities of aperient medicine, now rarely require it; and when it is needed, a smaller portion is found sufficient. This is not confined to the aged, for even in children I have witnessed a similar alteration in their former habits.

as cerebritis, hydrocephalus, pneumonia, enteritis, peritonitis, or hepatitis, it is an extremely inert and unjustifiable practice, to wait for twenty-four hours for the irritating effects of a blister, when the same may be produced in as many minutes by epithems of warm oil of turpentine.

Veterinary surgeons have condemned the external use of turpentine as an epispastic; it has been asserted that, when applied to the horse, it prevents the hair from growing. I do not think this correct. Some years ago I had a gray mare, which was seriously injured about the head and forelegs by an accident. Contrary to the recommendation of my veterinary surgeon, who insisted upon the application of tincture of myrrh, and greasy unguents containing gunpowder, I determined for once to try the experiment, if an injury to a horse might not be remedied by the same means as one in a human subject. I had the wounds carefully fomented and poulticed, and afterwards applied an ointment, consisting of resin ointment and oil of turpentine. The animal recovered without any material disfigurement. Last year I had a black horse consigned to me by a friend in Yorkshire, which met with a severe accident in its transit on the railway. The horse was treated in the same way as the one above, and in a few months was perfectly restored, without any other blemish.

The liniment, by means of which the celebrated quack St. John Long was supposed to have performed miraculous cures, was a mixture of the oil of turpentine, pyroligneous acid, and yolk of egg.*

As a *vermifuge*, turpentine has been given in the form of Chambert's oil. This is made by mixing one part of the empyreumatic oil of hartshorn, with three of oil of turpentine, allowing them to stand for three days, and afterwards distilling off three-fourths of the mixture by the aid of a sand bath. It very soon becomes blackened, by exposure to the air, and therefore ought to be kept well corked, and excluded from the light. It is extreme-

---

*This liniment is an excellent counter-irritant. We used it as an external stimulant in some cases of cholera during the past epidemic, as recommdnded by Dr. James Bird; and we frequently employ it as a counter-irritant in phthisis, and other chest diseases.—Editor.

ly nauseous; and, on that account, is not likely to come into general use.

As a *purgative*, turpentine ought never to be administered alone, in large doses, during the winter, or in cold damp weather: because under these circumstances, it tends, in common with other hydrocarbons, to supply fuel to the body for the evolution of animal heat, rather than exert any therapeutic property. Indeed, I very much question the propriety of giving it alone, as a purgative under any circumstances whatever. There are some writers who do not hesitate to recommend it in doses which I consider unjustifiable. In winter, cerebral congestion may supervene; in summer, intractable diarrhea, from over-excitement of the mucous membrane of the bowels. The case of Dr. Copland furnishes an instructive example on this head: ten drachms of the oil of turpentine were swallowed, and failed to induce action of the bowels or kidneys; the consequence was, high cerebral excitement, followed by a train of unpleasant symptoms, which it would be dangerous, in some constitutions, to excite.

Turpentine is, however, often a valuable addition to other purgatives, as it possesses the faculty of increasing their activity in a remarkable degree. I have known a lady, who, for forty years, was unable to procure an evacuation without the most drastic purgatives. She succeeded in obtaining daily action, by the simple combination of a teaspoonful of caster oil with ten drops of oil of turpentine. I have had another case under my care, where the same combination enabled me to relieve the augmented suffering occasioned by obstruction of the bowels from chronic meningo-myelitis of several years duration.

Whatever may be the object for which turpentine is exhibited as a purgative, whether for the expulsion of parasites infesting the human body, or as a revulsive in cerebral affections, the dose should never exceed half an ounce at one time; and to insure its purgative action, it ought to be united with some other aperient, as castor oil, compound infusion of senna, sulphate of magnesia, or the decoction of the bark of the root of the pomegranate. If prescribed in the above dose, in conjunction with any other active purgative, we run little risk of inducing strangury, or any other unpleasant symptom. It may be repeated at intervals of four hours, with perfect safety. Though some authors have stated that the dose of the oil of turpentine may be from half an ounce to two, or even four ounces he must be a very bold practitioner who would take this suggestion for his guide. If the first-named quantity will not suffice for the destruction and consequent expulsion of a tenia, a larger amount given at one time will equally fail; for it is not by the aperient properties alone of the medicine (as I shall hereafter show) that the death of the worm is effected.* As a *diuretic*, the dose may be from five to thirty drops, taken in any aromatic water, or mineral saline. I have rarely found patients object to its use, when exhibited with the salines of either Cheltenham or Harrowgate; and the numerous cases in which I have prescribed it, in conjunction with the waters from these mineral springs, have convinced me, that this union is especially indicated where we are anxious to direct its influence to the renal organs.

As an *astringent*, in doses varying from 20 minims to a drachm, according to the urgency of the symptoms, and repeated every three or four hours, turpentine is one of the most efficacious remedies which we possess. The best vehicle for its administration, in the first place, is water, flavored with syrup of orange, or any other agreeable aromatic. It may afterwards be advantageously combined with any other therapeutic agents, which the special nature of the case may require: thus, in epistaxis depending upon rupture of one or more small vessels, and where much arterial blood has been lost, muriated tincture of iron will form a valuable adjunt. In hemate-

---

*There may be special cases, but they will be extremely few, in which an extraordinary dose of any particular medicine may be peremptorily called for by the condition of the patient. For instance, I once gave to a man laboriug under delirium tremens, seven grains of the acetate of morphia, in divided doses, within two hours, ere I could allay the inordinate and convulsive movements, and restrain the shrieks of the wretched sufferer. Again, at another time, I exhibited to a female, in the presence of Dr. Logan, twelve ounces of sulphuric ether, when the principles of etherization were first introduced, and kept this woman in a state of insensibility for upwards of six hours. Although both these cases did well, they are exceptional ones, and ought never to be imitated, except in emergencies of the most urgent description.

mesis and other sanguineous discharges from the bowels, it may be united with compound infusion of roses, sulphate of magnesia, iced-water, and solutions of tannic or gallic acid. In some forms of hemoptysis, it may usefully be added to infusions of matico; in hematuria, to the decoctions of uva ursi, chimaphila, pyrola, etc.; or to tincture of sesquichloride of iron, etc. In purpura hemorrhagica, the decoctions or infusions of the barks form with it an excellent adjuvant. In hemoptysis, it has speedily and effectually arrested the hemorrhage; and is a much safer remedy than lead.

In my experience, there is no single medicine in the materia medica that can be compared with it as a *styptic*, either as to certainty of action or to the safety of its effects. It is compatible alike with acids and alkalies.

The *external use* of turpentine has been very general for a great number of years, alone or combined with other rubefacients, such as mustard, strong liquor ammonia, pyroligneous acid, cajeput oil, wine of hellebore, colchicum or opium, tartar emetic, croton oil, etc. It has very frequently been found of permanent utility, when applied as a warm epithem to the skin in pulmonary affections. Its action is twofold; first, it induces rapid though often transient counter-irritation; secondly, its vapor is inhaled into the lungs, and by its constringent operation on the extreme capillaries of the pulmonary texture, is not infrequently productive of great relief in some affections of these organs. For the purpose of inhalation, I am in the habit of dispersing its vapour through the room by evaporating water containing a portion of it, by the aid of a spirit lamp. When thus diffused through the atmosphere, it may be breathed for two or three hours in the course of the day, by the most delicate-chested person, and often with the most marked and striking amelioration of their pectoral symptoms.

Long after the patient has left the room, he is conscious of the taste and smell of the turpentine. I have often detected its presence some hours after he had been submitted to its penetrating influence. I have also employed camphine in the form of a bath, mixed with common soda; or two pounds of the latter with from a quarter of a pint to half a pint of camphine, and half an ounce of oil of rosemary, will form an excellent bath. In delicate skinned patients, females and children, ℥ii of camphine will be sufficient. I may remark, *in limine*, that the alkaline camphine bath possesses virtues peculiarly its own. In the coldest day in winter, as I have verified in more than one instance, it may be employed with the most perfect safety. Whilst the individual is in the bath, he experiences, to my knowledge, no disagreeable annoyance from the disengaged vapour; on the contrary, if we except the taste of the turpentine, which for some time remains in the mouth, a sense of calmness and tranquillity very often follows a previously disturbed, irregular, or excited condition of the respiratory or sanguiferous systems. After five minutes recumbency in the bath, the pulse is found to become fuller, softer, and slower; I have seen it fall from 100 to 80. The respiration also becomes freer, deeper, and less labored. On coming out of the bath, the whole skin has a peculiar velvety, soft, and agreeable feeling; the breath is strongly tainted with the terebinthinaceous odor. If it have not been too hot, a pleasurable tingling warmth is experienced throughout the whole cutaneous surface; and this, with the preceding symptoms, may continue twenty-four hours. One great advantage of this bath will be found in the circumstance, that it may be employed at a heat from 10 to 15 degrees below the temperature of the ordinary warm one, without inducing that sensation of chill to which some delicate constitutions are so peculiarly obnoxious; ten or fifteen minutes is the length of time a patient ought to remain in a bath of this description. In the first instance, it is well for patients to commence with a smaller quantity of the turpentine and soda, say a pound of the latter with two or three ounces of the former, and gradually increase its strength on each repetition of the bath, to the first-mentioned proportions. This bath may be taken every second or third day, according to the urgency of the symptoms and the nature of the affection for which it is prescribed.

I come now to a more particular enumeration of the maladies for which turpentine and its preparations have been chiefly recommended. They are—sanguineous exhalations from the mucous surfaces, epistaxis, hemoptysis, melena, purpura hemorrhagica;* consumption, chronic bronchitis, mucous or purulent discharges from the urethra;† grubs infesting the urethra, tenia, ascarides;‡ typhoid, yellow and puerperal fevers, plague;§ abdominal obstructions, strangulated hernia, tympanitis, colica pictonum, biliary concretions;‖ traumatic tetanus, trismus;¶ apoplexy, hydrocephalus, acute and chronic, epilepsy;** neuralgia, sciatica, rheumatism;†† diabetes, dropsy;‡‡ inflammations of the eye;§§ cholera, renal hydatids, suppression of urine;‖‖ burns, wounds, poisoning by prussic acid or opium, salivation.¶¶—*London Journal Medicine*, April, 1850.

*Adair, Brooke, Cheyne, Clutterbuck, Copland, Elliotson, Hunter, Mageé, Nichol (W.,) Thompson, Vincent, Younge.

†Aretæus, Celsus, Dioscorides, Van Swieten.

‡Birkbeck, Cross, Fenwick, Fothergill, Gomés, Hancock, Hartle, Kennedy, Knox, Laird, Lettsom, Maldon, Mello, Ozanam, Pereira, Saner, Winstone.

§Atkinson, Blundell, Brenan, Chapman, Copland, Cullen, Douglas, Farre, Faulkner (Sir A. Brooke,) Fernandez, Gooch, Hamilton, Holst, Johnson, Kinneir, Moran, Payne, Physick, Pritchard, Wood.

‖Boerhaave, Durand, Gibbon, Green, Guyton de Morveau, Hall (Marshall,) Hamilton (C. B.,) Kinglake, McWilliams, Odier, Paris, Ramsbotham, Sewell, Sprengel.

¶Gibbon, Hutchinson, Mott, Phillips.

**Latham, Lithgow, Money, Moran, Percival. Pritchard, Young.

††Bonnet, Cheyne, Ducros, Dufour, England, Hild, Home, La Roque, Lenton, Martinet, Maton, Pitcairn, Recamier, Thilenius.

‡‡Darwin, Werlhoff.

§§Burke, Carmichael, Foote, Guthrie, Hynam, Langier, Middlemore, Wright.

‖‖Bayle, Copland, Neale, Pereira.

¶¶Emmert, Geddings, Hanold, Heister, Jenkins, Kentish, King, Orfila, Paré (Ambroise,) Percy, Pott.

